# Mixed exocrine-neuroendocrine intraductal papillary mucinous neoplasms of the pancreas: a case series

**DOI:** 10.1093/omcr/omag147

**Published:** 2026-09-02

**Authors:** Jaimin P Chauhan, Aryan K Sharma, Abed M Zaitoun, Dileep N Lobo

**Affiliations:** University of Nottingham Medical School, Nottingham University Hospitals NHS Trust, Queen’s Medical Centre, Derby Road, Nottingham NG7 2UH, Nottinghamshire, United Kingdom; University of Nottingham Medical School, Nottingham University Hospitals NHS Trust, Queen’s Medical Centre, Derby Road, Nottingham NG7 2UH, Nottinghamshire, United Kingdom; Department of Cellular Pathology, Nottingham University Hospitals NHS Trust, Queen’s Medical Centre, Derby Road, Nottingham NG7 2UH, Nottinghamshire, United Kingdom; Nottingham Digestive Diseases Centre, Division of Translational Medical Sciences, School of Medicine, University of Nottingham, Queen’s Medical Centre, Derby Road, Nottingham NG7 2UH, Nottinghamshire, United Kingdom; Nottingham Digestive Diseases Centre, Division of Translational Medical Sciences, School of Medicine, University of Nottingham, Queen’s Medical Centre, Derby Road, Nottingham NG7 2UH, Nottinghamshire, United Kingdom; Department of Surgery, Nottingham University Hospitals NHS Trust, Queen’s Medical Centre, Derby Road, Nottingham NG7 2UH, Nottinghamshire, United Kingdom; National Institute for Health Research Nottingham Biomedical Research Centre, Nottingham University Hospitals NHS Trust and University of Nottingham, Queen’s Medical Centre, Derby Road, Nottingham NG7 2UH, Nottinghamshire, United Kingdom; MRC Versus Arthritis Centre for Musculoskeletal Ageing Research, School of Life Sciences, University of Nottingham, Queen’s Medical Centre, Derby Road, Nottingham NG7 2UH, Nottinghamshire, United Kingdom; Division of Surgery, Perelman School of Medicine, University of Pennsylvania, Spruce Street, Philadelphia 19104, PA, United States

**Keywords:** immunohistochemistry, intraductal papillary mucinous neoplasm, mixed exocrine-neuroendocrine tumour, mucin gene, neuroendocrine tumours, pancreatic cystic lesions

## Abstract

We retrospectively reviewed histopathological and immunohistochemical data from patients with mixed exocrine-neuroendocrine intraductal papillary mucinous neoplasms (IPMNs) of the pancreas identified at a single centre between December 2023 and June 2026. Surgical specimens underwent detailed evaluation, including mucin profiling (MUC1, MUC2, MUC5AC) and neuroendocrine markers (synaptophysin, chromogranin A). Ten patients (median age 68.5 years, range: 31–78 years; 6 male) presented mainly with abdominal pain, weight loss, or jaundice. Most lesions were intestinal type IPMNs with focal or interspersed neuroendocrine components. Mucin expression patterns aligned with established subtype profiles, and neuroendocrine differentiation was confirmed immunohistochemically. Only one of the lesions met the World Health Organization (WHO) criteria for mixed neuroendocrine-non-neuroendocrine neoplasms (MiNEN) due to the non-invasive IPMN component. This case series highlights a distinct subset of mixed non-invasive IPMN-neuroendocrine tumours not currently recognized in WHO classifications. We propose recognition of this entity to improve diagnostic precision and guide management.

## Introduction

Intraductal papillary mucinous neoplasms (IPMNs) are mucin-producing cystic lesions of the pancreas arising from papillary proliferation of stem-like epithelial cells within the pancreatic ductal system [1]. They are the most common pancreatic cystic neoplasm and are estimated to account for approximately 80% of all pancreatic cystic lesions in the general population. Their true prevalence is likely underestimated as the majority remain clinically silent and are often identified incidentally during imaging performed for unrelated indications [2, 3]. IPMNs characteristically secrete large quantities of mucin, leading to cystic dilatation of the pancreatic ducts, and carry a risk of progression from benign intraductal neoplasia to invasive ductal adenocarcinoma [4]. Tumour grading is based on the degree of dysplasia (low-grade, high-grade, or invasive), with estimated progression to adenocarcinoma typically occurring over 5–6 years [2].

IPMNs are the second most common subtype of pancreatic exocrine neoplasms and are classified histologically into gastric, intestinal and pancreatobiliary types [5–7]. Exocrine pancreatic neoplasms represent up to 95% of pancreatic cancers whereas neuroendocrine tumours make up only a small proportion (<5%) [8].

Although rare, mixed exocrine-neuroendocrine tumours have been reported in the pancreas. In particular, some IPMNs have demonstrated neuroendocrine differentiation, as evidenced by immunohistochemical positivity for synaptophysin and chromogranin A [9]. These may occur as composite tumours, where exocrine and endocrine elements are intermingled, or as collision tumours with two spatially distinct components. According to the World Health Organization (WHO) classification, neoplasms composed of both neuroendocrine and non-neuroendocrine cancer components, each accounting for at least 30% of the tumour volume, are classified as mixed neuroendocrine-non-neuroendocrine neoplasms (MiNENs) [7]. Pancreatic MiNENs are most commonly composed of invasive ductal adenocarcinoma and high-grade neuroendocrine carcinoma, referred to as adeno-MiNEN [10].

However, in cases involving IPMN with well-differentiated neuroendocrine tumour (NET) components, the classification becomes more complex and less clearly defined. While these mixed lesions deviate from the classic IPMN subtypes, they do not always meet the strict WHO criteria for MiNENs, particularly when the IPMN component is non-invasive and/or the neuroendocrine component constitutes less than 30% of the lesion [7]. Furthermore, the presence of a well-differentiated NET, rather than a high-grade neuroendocrine carcinoma, might not carry the same prognostic or therapeutic implications as classical MiNENs.

Recent molecular studies have identified shared driver mutations (specific DNA changes in cancer cells that provide a survival or growth advantage, actively promoting tumour development), including Kirsten rat sarcoma viral oncogene homolog (KRAS) and Guanine Nucleotide-binding protein, Alpha Stimulating (GNAS), in both IPMN and associated neuroendocrine components. These findings challenge the traditional view that the neuroendocrine component necessarily represents a separate neoplastic entity, suggesting instead that neuroendocrine differentiation can arise as part of the natural progression of IPMNs [6, 11].

The aim of this study was to describe the clinicopathological features of mixed exocrine-neuroendocrine IPMNs.

## Methods

We performed a review of the histological database maintained by the Department of Histopathology at Nottingham University Hospitals NHS Trust, Queen’s Medical Centre, between December 2023 and June 2026. Using the Systematized Nomenclature of Medicine—Clinical Terms (SNOMED—CT) classification system, we identified cases of IPMN with mixed exocrine-neuroendocrine features. Demographic data, clinical presentation, type of surgery and histopathological details were recorded. All immunohistochemical (IHC) staining was done in our department; these included MUC1, MUC2, MUC5AC, MUC4, CK7, CDX2, P16, Synaptophysin, and Chromogranin A. The Ki67 proliferative marker was used on epithelial elements. The intensity of immunohistochemical staining was graded on a scale ranging from G0 (no stain uptake) to G3 (strong stain uptake). The extent of staining across each sample was further categorised as focal, moderate, or diffuse.

Ethics approval was obtained from the UK Health Research Authority Ethics Committee for the use of banked tissue (IRAS Project ID: 233124; Sponsor amendment reference: 17060 NSA02). The need for obtaining consent from patients was waived.

## Results

A total of 10 patients with mixed exocrine-neuroendocrine IPMNs were identified from the histological database. The patients’ demographic, clinical, radiological, operative and histopathological characteristics, along with follow up details are summarised in Table 1.

**Table 1:** Demographics, presenting complaints, imaging findings, surgical procedures, diagnoses, IPMN histological classification and postoperative follow up.

Case	Age (years)/Sex	Past history	Clinical presentation	Radiological findings	Surgical procedure	Diagnosis	IPMN histological classification	Follow up
1	31 Male	Acute pancreatitis	Abdominal pain	MRCP: growth of a known side branch IPMN in the uncinate process and head of pancreas, increasing from 2.4 cm to 3.8 cm, with 6 mm main pancreatic duct dilatation	Pancreaticoduodenectomy	IPMN	Mixed endocrine and exocrine IPMN demonstrating predominantly intestinal exocrine features	No adjuvant treatment. Alive 29 months after surgery
2	64 Male	Nil	Weight loss, early satiety, palpable liver edge, and obstructive jaundice	CT/MRI: dilation of the biliary tree, to the level of the pancreas, accompanied by dilation of the main pancreatic duct. Evidence of mild compression of the superior mesenteric vein, at its confluence with the portal vein	Pancreaticoduodenectomy	Pancreatic adenocarcinoma arising from a background of IPMN	Mixed endocrine and exocrine IPMN demonstrating predominantly intestinal exocrine features	Developed local recurrence and metastatic disease (from pancreatic adenocarcinoma) at 12 months. Treated with chemotherapy. Alive 25 months after surgery
3	64 Male	Nil	Weight loss	CT: poorly enhancing, ill-defined lesion in the head and uncinate process of pancreas	Pancreaticoduodenectomy	IPMN	Mixed endocrine and exocrine IPMN demonstrating predominantly intestinal exocrine features	No adjuvant treatment. Alive 31 months after surgery
4	73 Male	Nil	Abdominal pain	CT/MRI: growing pancreatic head cyst associated with IPMN, causing dilation of the main pancreatic duct	Pancreaticoduodenectomy	IPMN	Mixed endocrine and exocrine IPMN demonstrating predominantly intestinal exocrine features	No adjuvant treatment. Alive 26 months after surgery
5	71 Female	Nil	Abdominal pain	CT: 2.5 cm lesion in the body of the pancreas MRCP: complex cystic mass in the body of the pancreas	Distal pancreatectomy and splenectomy	Pancreatic serous cystadenoma and side branch IPMN	Mixed endocrine and exocrine IPMN demonstrating predominantly pancreatic exocrine features	No adjuvant treatment. Alive 21 months after surgery
6	56 Female	Metastatic caecal cancer	Incidental finding on post-SABR imaging for pulmonary metastasis from caecal cancer	Imaging: two suspicious lesions in the pancreatic tail FDG-CT: increased uptake of FDG in the lesions. Thought to be metastatic lesions from caecal primary	Initial 6 cycles of chemotherapy for metastatic colorectal cancer Distal pancreatectomy and splenectomy	Primary pancreatic ductal adenocarcinoma, intestinal sub-type, arising from a background of intestinal subtype IPMN	Mixed endocrine and exocrine IPMN demonstrating predominantly intestinal exocrine features	Received adjuvant chemotherapy for pancreatic cancer. Alive 21 months after surgery
7	78 Female	Nil	Lethargy, reduced appetite, weight loss	CT: large hypervascular mass involving the pancreas. Follow-up imaging post-chemotherapy: growth of mass, encasing the splenic artery, occluding the splenic vein and abutting the stomach and left adrenal gland EUS-guided FNA consistent with neuroendocrine tumour	Initial 6 cycles of chemotherapy with capecitabine and temozolamide Subsequent distal pancreatectomy, splenectomy, left adrenalectomy and gastric sleeve resection	Pancreatic neuroendocrine tumour with hepatoid and phaeochromocytoma like morphology. The neuroendocrine tumour was associated with incidental side branch IPMN	Mixed endocrine and exocrine IPMN demonstrating predominantly pancreatic exocrine features	No adjuvant treatment. Alive 19 months after surgery
8	67 Male	Non-Hodgkin’s lymphoma	Lethargy	EUS: 22 mm hypoechoic mass in the tail of pancreas, abutting the splenic vessels	Distal pancreatectomy and splenectomy	Adenosquamous IPMN, with mixed exocrine and endocrine features	Mixed endocrine and exocrine IPMN demonstrating predominantly pancreatic exocrine features	No adjuvant treatment. Alive 17 months after surgery
9	71 Female	Nil	Abdominal pain	CT: pancreatic cyst in the head and uncinate process of pancreas, measuring 34 × 40 mm EUS: thin-walled cyst with single septation, demonstrating string sign	Pancreaticoduodenectomy	IPMN with low-grade dysplasia and mixed exocrine and endocrine features	Mixed endocrine and exocrine IPMN demonstrating predominantly pancreatico-gastric exocrine features	No adjuvant treatment. Alive 5 months after surgery
10	70 Male	Previous transitional cell carcinoma of the bladder	Incidental finding on follow-up CT	PET-CT showed a 52 × 34 mm bilobar mass in the tail of the pancreas with intense FDG uptake. Mass previously seen on CT and MRI EUS guided FNA: moderately differentiated adenocarcinoma	Distal pancreatectomy and splenectomy	Pancreatic ductal adenocarcinoma. IPMN with low-grade and high-grade dysplasia and mixed exocrine and endocrine features	Mixed endocrine and exocrine IPMN demonstrating predominantly pancreatico-gastric exocrine features with focal intestinal type. As there was adenocarcinoma with neuroendocrine differentiation, this fulfilled the diagnostic criteria for MiNEN	Awaiting adjuvant chemotherapy. Alive 1 month after surgery

CT = computed tomography, ERCP = endoscopic retrograde cholangiopancreaticography; EUS = endoscopic ultrasound; FDG = fluorodeoxyglucose; FNA = fine needle aspiration; IPMN = intraductal papillary mucinous neoplasm; MRCP = magnetic resonance cholangiopancreaticography; MiNEN = mixed neuroendocrine-non-neuroendocrine neoplasm; MRI = magnetic resonance imaging; PET = positron emission tomography; SABR = stereotactic ablative radiotherapy

The median age was 68.5 (range: 31–78) years, with a slight male predominance (n = 6, 60%). The most common presenting symptoms were abdominal pain, weight loss, lethargy and obstructive jaundice. Notably, two patients were diagnosed incidentally: one during post stereotactic ablative radiotherapy (SABR) imaging for pulmonary metastases from a previously resected caecal adenocarcinoma (Case 6) and the other during annual surveillance imaging for transitional cell carcinoma of the bladder (Case 10). The first underwent chemotherapy (FOLFOX—folinic acid, 5-fluouracil and oxaliplatin) for metastatic colorectal cancer after the pancreatic lesions were identified, achieving a reduction in lesion size; surgical resection was subsequently performed due to slow disease progression. However, postoperative histology revealed a primary pancreatic ductal adenocarcinoma. The location of radiologically identified lesions varied, with four patients having a lesion that spanned two pancreatic regions. The most frequently affected sites were the head (n = 4) and tail (n = 5), followed by the uncinate process (n = 3) and body (n = 3) of the pancreas. Imaging also identified either main pancreatic duct or common bile duct dilatation in three patients. All patients underwent surgical intervention: Whipple’s procedure (pancreaticoduodenectomy) in five and distal pancreatectomy with splenectomy in five.

**Figure 1:**
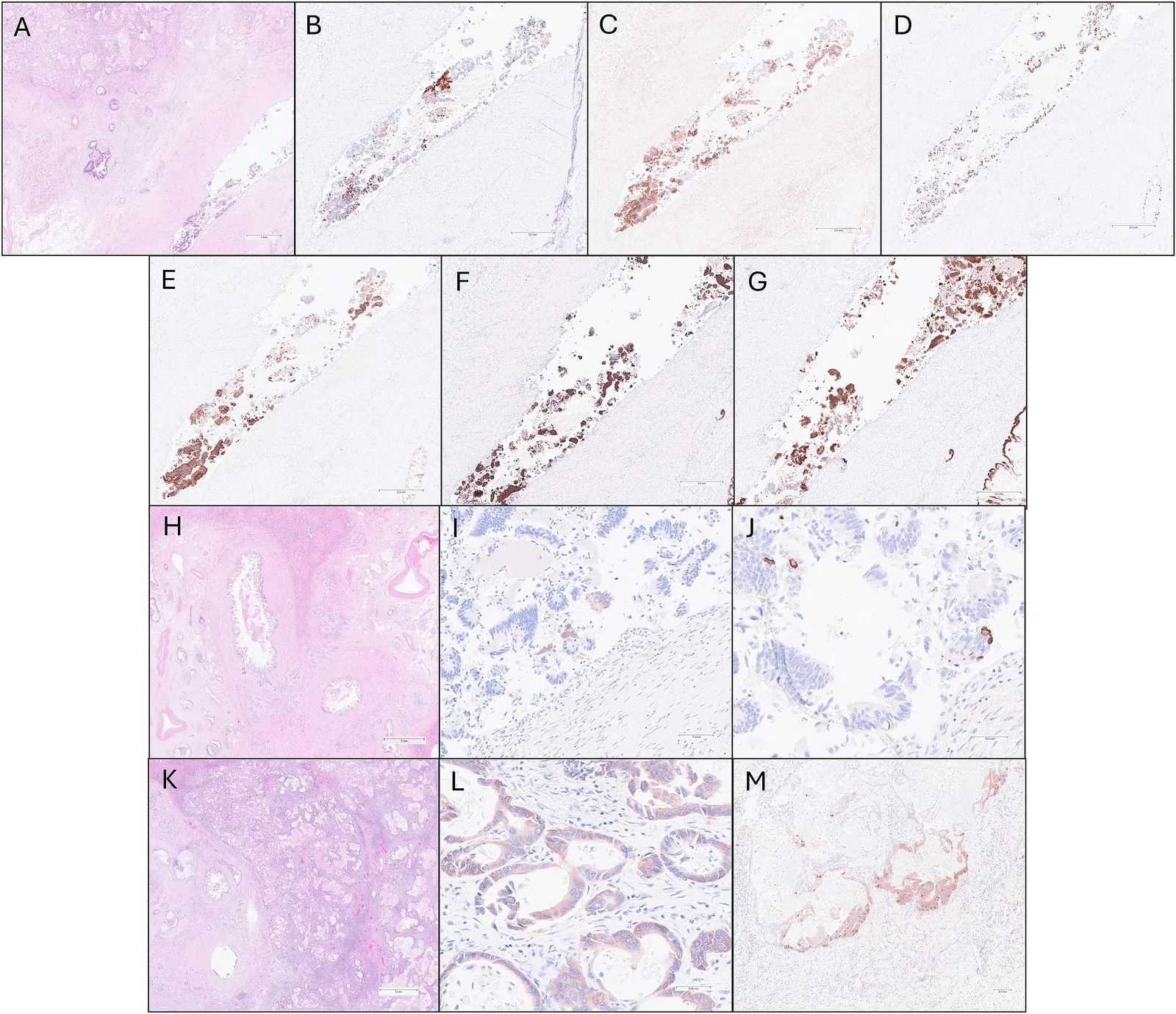
Images A-G show staining of a mixed exocrine-neuroendocrine IPMN, demonstrating predominantly intestinal exocrine features. A) Illustrates the sample stained with haematoxylin and eosin (H & E × 2). Immunohistochemical (IHC) staining was performed for: B) MUC2 (×5) [G2 moderate], C) epithelial membrane antigen (EMA × 5) [G2 diffuse], D) Ki67 (×5) [G3 moderate], E) CK7 (×5) [G2, diffuse], F) CK20 (×5) [G3 diffuse], G) CDX2 (×5) [G3 diffuse]. Images H-J show further IHC staining of the IPMN. H) H & E (×2), I) synaptophysin (×20) [G1 focal], J) chromogranin A (×40) [G3 focal]. Images K-M show IHC staining of the associated invasive carcinoma. K) H & E (×2), L) synaptophysin (×40) [G1 diffuse], M) chromogranin a (×20) [G2 moderate].

**Figure 2:**
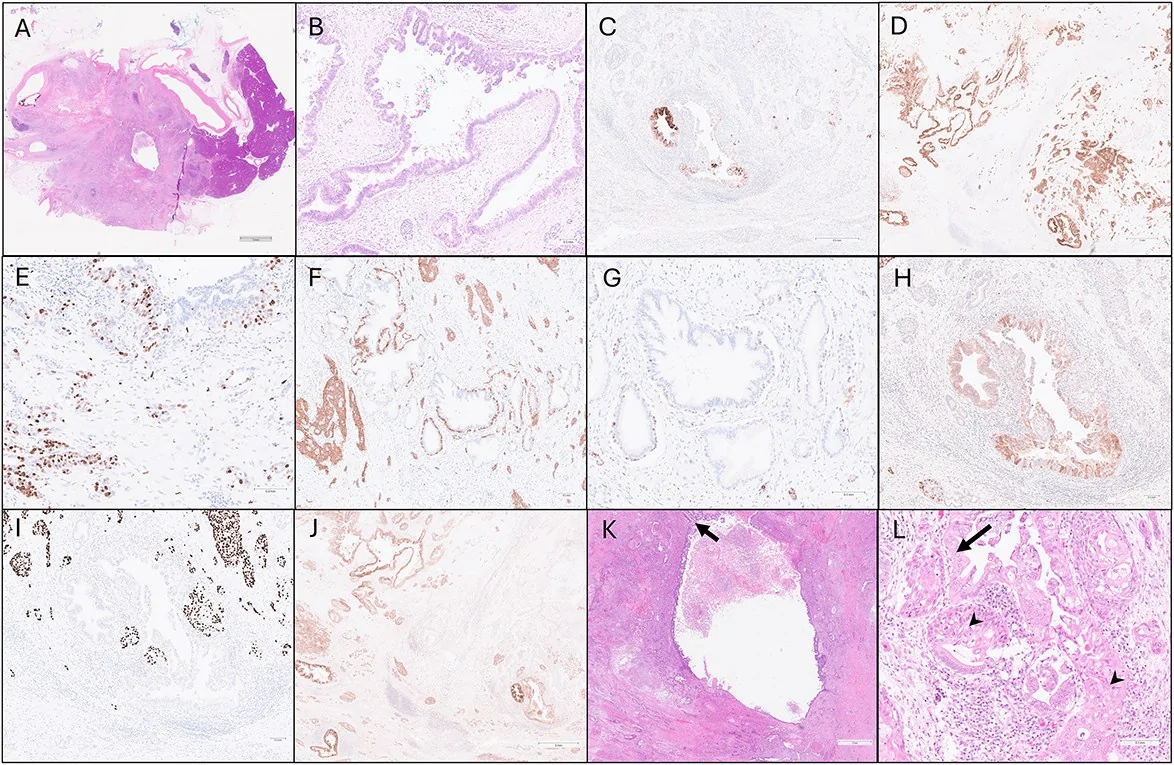
Staining of a mixed exocrine-neuroendocrine IPMN, demonstrating predominantly pancreatic exocrine features. A) Illustrates the sample stained with haematoxylin and eosin (H & E × 0.4). B) H & E (×10). Immunohistochemical (IHC) staining was performed for: C) MUC5AC (×5) [G3 moderate], D) epithelial membrane antigen (EMA × 2) [G2 diffuse], E) KI67 (×20) [G3 diffuse, > 20%], F) synaptophysin (×10) [G3 moderate], G) chromogranin A (×20) [G2 focal], H) P16 (×10) [G2 moderate], I) P40 (×10) [G3 diffuse], J) BEREP4 (×2) [G2 moderate], K) H & E (×2) demonstrating glandular epithelial changes (arrow) within the IPMN, L) H & E (×20) demonstrating glandular (arrow) and squamous (arrow heads) features within the IPMN.

**Figure 3:**
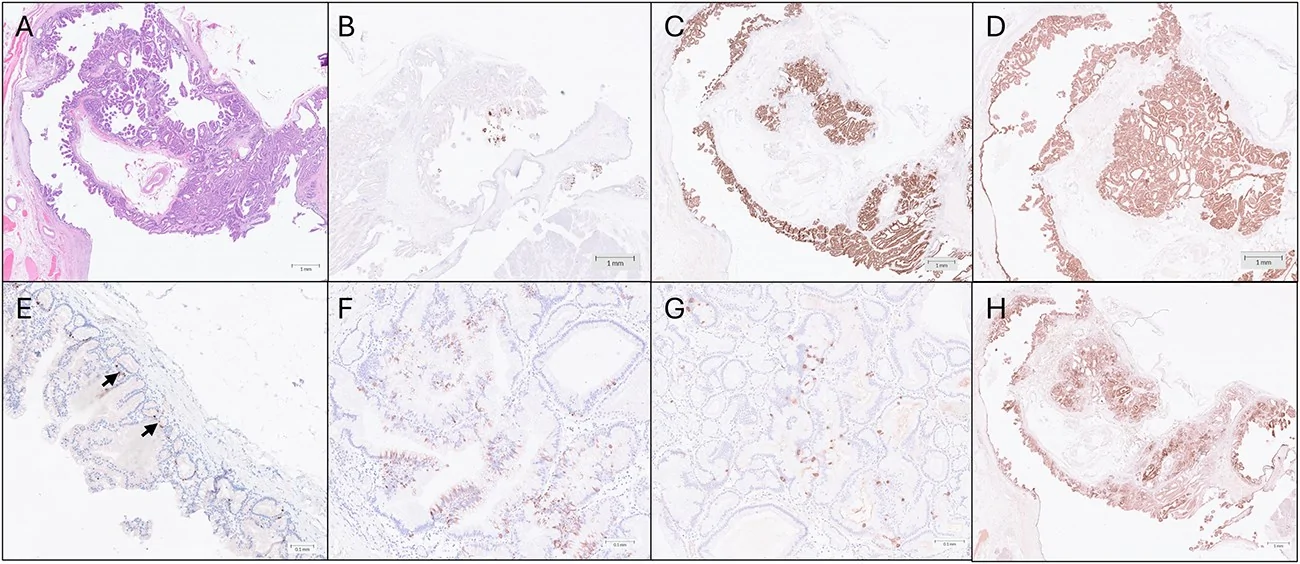
Staining images of mixed endocrine and exocrine IPMN, demonstrating predominantly pancreaticobiliary and gastric exocrine features. A) Illustrates the sample stained with haematoxylin and eosin (H & E × 1). IHC staining was performed for: B) MUC2 (×1) [G2 focal], C) MUC5AC (×1) [G3 diffuse], D) epithelial membrane antigen (EMA × 1) [G3 diffuse), E) Ki67 with arrows indicating positivity (×15) [G3 focal, < 2%]), F) synaptophysin (×15) [G2 focal], G) chromogranin A (×15) [G1 focal], H) P16 (×1) [G3 diffuse].

**Figure 4:**
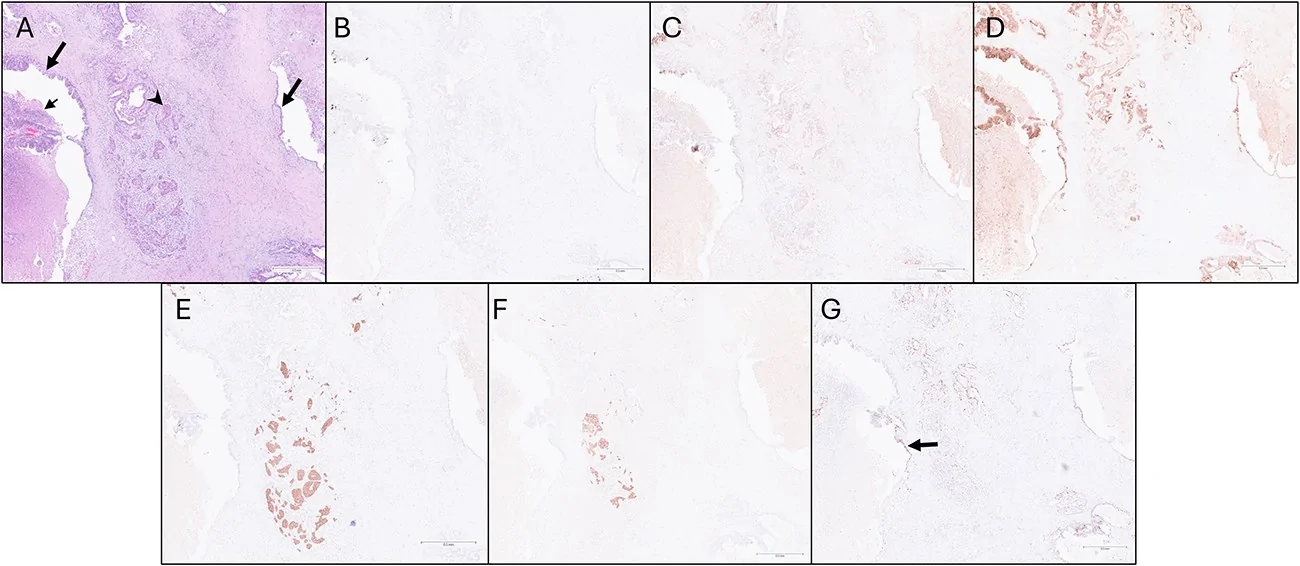
Staining images of mixed endocrine and exocrine IPMN demonstrating predominantly pancreatico-gastric exocrine features with focal intestinal type. A) Illustrates the sample stained with haematoxylin and eosin (H & E × 5); the long arrows represent the IPMN, the short arrow represents tufting within the IPMN and the arrowhead is the invasive component. IHC staining was performed for: B) MUC2 (×5) [G2 focal], C) MUC5AC (×5) [G1 moderate], D) epithelial membrane antigen (EMA × 5) [G3 diffuse), E) synaptophysin (×5) [G2 focal], F) chromogranin A × 5) [G1 focal], G) Ki67 with the arrow indicating positivity (×5) [G2 moderate, 20%]).

Histopathology confirmed the predominant exocrine subtype of our cases of mixed exocrine-neuroendocrine IPMNs. The most frequent histological subtype was intestinal (n = 5), followed by pancreatic (n = 3), and pancreatico-gastric (n = 2) subtype. Examples of each IPMN subtypes can be seen in Figs. 1-4. A summary of the IHC analysis is provided in Table 2. Accurate assessment for the endocrine differentiation was difficult due to the focality of endocrine cells in the stained section. Hence, it was not possible to provide a Ki-67 index for the endocrine component. Three patients (Cases 2, 6 and 10) had associated pancreatic ductal adenocarcinoma. This diagnosis was made preoperatively in one (Case 10) and in the other (Case 6) the preoperative cytology suggested metastatic colorectal adenocarcinoma, but the postresection histopathological diagnosis was pancreatic ductal adenocarcinoma. A third patient (Case 2) was found to have an incidental pancreatic ductal adenocarcinoma on postoperative histology. MiNEN was diagnosed in only one patient (Case 10) as the criteria for MiNEN were not fulfilled in the other two patients.

**Table 2:** Immunohistochemistry findings.

Case No.	Mixed IPMN predominant exocrine subtype	MUC1 (EMA)	MUC2	MUC5AC	MUC4	CK7	CK20	CDX2	P16	Synapto-physin	Chromo-granin A	Ki-67
1	Intestinal	+	+	+	+	+	+	+	+	+	+	+
2	Intestinal	+	+	+	N/D	N/D	N/D	N/D	+	+	+	+
3	Intestinal	+	−	+	N/D	+	N/D	N/D	+	+	+	+
4	Intestinal	+	+	+	N/D	+	N/D	N/D	+	+	+	+
5	Pancreatic	+	N/D	+	N/D	N/D	N/D	N/D	N/D	+	N/D	+
6	Intestinal	+	+	−	N/D	+	+	N/D	+	+	+	+
7	Pancreatic	+	N/D	+	N/D	N/D	N/D	N/D	N/D	−	N/D	N/D
8	Pancreatic	+	N/D	+	N/D	N/D	N/D	N/D	+	+	+	+
9	Pancreatico-gastric	+	+	+	+	+	N/D	+	+	+	+	+
10	Pancreatico-gastric	+	+	+	+	+	+	+	N/D	+	+	+

‘N/D’ indicates that IHC staining for this marker was not performed on the corresponding sample.

EMA = epithelial membrane antigen

All patients were alive during the median follow up period of 21 months (range 1–31 months) from the date of the operation (Table 1).

## Discussion

Our centre has previously reported three cases of this rare entity in 2013 [12] and we are now identifying this lesion more frequently. Most lesions in this series of IPMNs with mixed exocrine-neuroendocrine differentiation demonstrated predominantly intestinal type exocrine features, with neuroendocrine components identified either focally or interspersed throughout the tumour. This hybrid morphology adds complexity to the diagnostic process, particularly in the context of evolving classification systems.

The value of IHC in the diagnosis and subtyping of IPMNs is well established. In particular, mucin gene expression has been instrumental in classifying IPMN subtypes and estimating their malignant potential [13]. Mucins are high molecular weight, heavily glycosylated glycoproteins expressed in the epithelial cells of glandular tissues, where they play critical roles in maintaining epithelial homeostasis and contributing to carcinogenesis [14].

Among the most clinically relevant mucins, MUC1 is characteristic of the pancreaticobiliary subtype and is associated with high-grade dysplasia, aggressive, invasive behaviour and, thus, a poor prognosis. In normal tissues, MUC1 primarily functions as a protective physical barrier, shielding the apical cell membrane from mechanical and enzymatic damage [15, 16]. However, when mutated, MUC1 contributes to oncogenesis by promoting immune evasion, loss of cell polarity, and epithelial-mesenchymal transition [17]. MUC1 expression is generally uncommon among IPMNs exhibiting a specific exocrine subtype, such as pancreatic. However, MUC1 positivity is more frequently observed in IPMNs with mixed histologic features. In our series, MUC1 was expressed across all our cases, varying in distribution and intensity of staining.

Conversely, MUC2 expression, a hallmark of the intestinal subtype, is often associated with a more indolent clinical course in non-invasive IPMNs [18]. Unlike MUC1, it is rarely expressed in pancreatic intraepithelial neoplasms or conventional pancreatic ductal adenocarcinomas [19]. Notably, MUC2 was strongly expressed in the majority of our intestinal type IPMNs, aligning with existing literature and suggesting a lower overall risk of invasion in these cases.

MUC5AC was widely expressed in our cohort and, as suggested in previous studies, may serve as an early marker of neoplastic transformation; however, its association with tumour invasiveness appears to be weak or inconsistent [20, 21]. Among IPMN variants, the gastric type, is the most common subtype (comprising approximately 60–70% of cases) and are typically MUC1- and MUC2-negative with branch duct involvement with low grade-dysplasia [22, 23]. The intestinal type is generally MUC1-negative and MUC2-positive [14]. Our findings were consistent with these established patterns: most intestinal type cases in our cohort were MUC5AC- and MUC2-positive.

The diagnostic framework for IPMNs has evolved considerably over time. First described in the 1980s in Japan, IPMNs were formally distinguished from other mucinous pancreatic lesions by the WHO in the late 1990s [24]. The 2010 WHO classification introduced four histological subtypes (gastric, intestinal, pancreaticobiliary, and oncocytic) based on morphology and mucin expression profiles [25]. More recently, the 2019 WHO update reclassified the oncocytic subtype as a separate entity, intraductal oncocytic papillary neoplasm (IOPN), acknowledging its distinct histopathological and molecular features [7].

While the 2019 WHO revision introduced MiNENs, which reflect a more inclusive categorisation, only one case (Case 10) in our series fulfilled these criteria whilst the remainder did not due to the non-invasive nature of the IPMN component [7]. As such, they remain unclassified within the current WHO guidelines. Given the presence of both non-invasive exocrine and neuroendocrine elements, we propose a new classification: mixed non-invasive IPMN-neuroendocrine tumour. This category would accommodate tumours with dual differentiation that do not meet MiNEN criteria but nonetheless represent a distinct morphological and clinical entity. Although sporadic case reports have documented such occurrences, the current body of literature remains highly limited. Consequently, there is a lack of established guidelines regarding diagnostic and treatment pathways.

The strengths of our study include the extensive use of immunohistochemistry to characterise this subset of IPMNs with mixed exocrine-neuroendocrine differentiation. Although this represents one of the largest reported case series of such lesions, the overall sample size remains limited, reflecting the rarity of the entity. Further studies are required to validate these findings, including more comprehensive molecular analysis such as KRAS profiling. In addition, comparison with MiNENs and assessment of survival outcomes are constrained by the small number of cases with invasive carcinoma (n = 3).

This study expands on the limited literature by providing detailed, systematic IHC and mucin profiling of mixed exocrine-neuroendocrine IPMNs. Importantly, we have focused on non-invasive mixed lesions that fall outside current WHO classification criteria, thereby identifying a gap in existing frameworks and supporting recognition of this pattern as a distinct entity.

## Conclusion

Our case series highlights a subset of pancreatic neoplasms with mixed exocrine-neuroendocrine differentiation where the majority did not conform to current WHO classification criteria, given their non-invasive component. These lesions fall outside the definition of MiNENs, yet represent a distinct clinical and pathological entity. We propose recognising this category, provisionally termed ‘mixed non-invasive IPMN-neuroendocrine tumour’ to enhance diagnostic clarity and guide future management. Continued investigation is essential to characterise their molecular profile, natural history, and optimal therapeutic approach.

## Data sharing statement

Data will be available from AMZ (a.zaitoun@nhs.net) upon reasonable request.
